# Transfer of training from one working memory task to another: behavioural and neural evidence

**DOI:** 10.3389/fnsys.2015.00086

**Published:** 2015-06-02

**Authors:** Erin L. Beatty, Marie-Eve Jobidon, Fethi Bouak, Ann Nakashima, Ingrid Smith, Quan Lam, Kristen Blackler, Bob Cheung, Oshin Vartanian

**Affiliations:** ^1^Defence Research and Development Canada – Toronto Research CentreToronto, ON, Canada; ^2^Department of Psychology, University of Toronto ScarboroughToronto, ON, Canada

**Keywords:** working memory, n-back, cognitive training, delayed matching-to-sample, prefrontal cortex

## Abstract

N-back working memory (WM) tasks necessitate the maintenance and updating of dynamic rehearsal sets during performance. The delayed matching-to-sample (dMTS) task is another WM task, which in turn involves the encoding, maintenance, and retrieval of stimulus representations in sequential order. Because both n-back and dMTS engage WM function, we hypothesized that compared to a control task not taxing WM, training on the n-back task would be associated with better performance on dMTS by virtue of training a shared mental capacity. We tested this hypothesis by randomly assigning subjects (*N* = 43) to train on either the n-back (including 2-back and 3-back levels) or an active control task. Following training, dMTS was administered in the fMRI scanner. The n-back group performed marginally better than the active control group on dMTS. In addition, although the n-back group improved more on the less difficult 2-back level than the more difficult 3-back level across training sessions, it was improvement on the 3-back level that accounted for 21% of the variance in dMTS performance. For the control group, improvement in training across sessions was unrelated to dMTS performance. At the neural level, greater activation in the left inferior frontal gyrus, right posterior parietal cortex, and the cerebellum distinguished the n-back group from the control group in the maintenance phase of dMTS. Degree of improvement on the 3-back level across training sessions was correlated with activation in right lateral prefrontal and motor cortices in the maintenance phase of dMTS. Our results suggest that although n-back training is more likely to improve performance in easier blocks, it is improvement in more difficult blocks that is predictive of performance on a target task drawing on WM. In addition, the extent to which training on a task can transfer to another task is likely due to the engagement of shared cognitive capacities and underlying neural substrates—in this case WM.

## Introduction

Working memory (WM) can be defined as “a multicomponent system for active maintenance of information in the face of ongoing processing and/or distraction” (Conway et al., 2005, p. 770). Recently, there has been great theoretical and applied interest in the prospects of WM training for improving cognition. This interest stems from the possibility that improvements in WM performance as a function of training might be transferable to other mental activities similarly drawing on WM capacity (Klingberg, 2010; Morrison and Chein, 2011; Buschkuehl et al., 2012). Although there is evidence to show that WM training can produce improvements in verbal as well as visuospatial WM, reliable evidence regarding far transfer to untrained tasks is presently lacking (for review see Melby-Lervåg and Hulme, 2013).

An important factor that might affect transfer is the goodness-of-fit between the specific capacity enhanced during training and the cognitive requirements of the untrained activity. For example, Harrison et al. (2013) showed that training on simple and complex WM span tasks led to improved performance on similar tasks (i.e., reading span and rotation span), despite the use of material with different surface features. Thus, structural and functional similarities between the trained and untrained tasks (e.g., both necessitate the suppression of distractors) appear to increase likelihood of transfer. The same conclusion can be drawn from the study conducted by Dahlin et al. (2008) who demonstrated transfer to a test of WM (i.e., letter memory) after 5 weeks of training in a specific aspect of WM—updating. The control group did not receive any training or specific activity. Importantly, using functional magnetic resonance imaging (fMRI), the researchers were also able to determine that the transfer effect was based on a joint training-related increase in brain activation in the trained and target tasks in the striatum. No transfer was observed to the Stroop task—a task that does not involve updating, and does not typically engage the striatum. Dahlin et al.’s (2008) results suggest that to obtain transfer, it is necessary to train specific aspects of WM (e.g., updating) that are functionally shared by the trained and target tasks. In turn, likelihood of transfer is increased to the extent that training-related changes in neural function occur in the same brain region recruited in relation to the trained process (e.g., updating) in both tasks.

Consistent with these process-specific findings, Salminen et al. (2012) examined transfer effects from WM training to executive functions. Importantly, they mapped particular cognitive processes engaged by their WM training task (i.e., dual n-back) to four aspects of executive functions, and measured transfer effects separately for each of those four processes: updating, coordination of concurrent performance, task switching, and attention. Their results demonstrated transfer from WM training to all aspects of executive function except coordination of concurrent performance, which the authors attributed to a “lack of commonalities” between the trained and target tasks (e.g., differences in the extent to which speeded processing was necessary for optimal performance). Salminen et al.’s (2012) results reinforce the notion that transfer effects depend on specific cognitive processes shared by the WM training and target tasks (see also Persson et al., 2007; Karbach and Kray, 2009; Sprenger et al., 2013; Salminen et al., 2015).

Building on the idea that shared capacities increase the likelihood of transfer of training, we conducted a study to test the hypothesis that training on one WM task would be more strongly associated with better performance on another WM task than training on a task that does not tax WM function. Our training task consisted of the n-back task—one of the most commonly used tasks to assess WM performance in the cognitive neuroscience literature (Kane and Engle, 2002). The n-back task requires that participants decide, on a trial-by-trial basis, whether a stimulus presented in the current trial matches a target stimulus presented a specific number of trials earlier in the sequence. The letter *n* denotes the specific number of trials that separate the current trial from the target trial. This task necessitates both maintenance and updating of dynamic rehearsal sets during performance (Kane et al., 2007). In contrast, participants in the active control group completed the 4-choice reaction time (RT) task (Dollins et al., 1993), which consists of pressing one of four buttons as quickly as possible when one of four target locations on a screen is highlighted (each target being matched to a given button). This task is not hypothesized to tax WM function.

**FIGURE 1:**
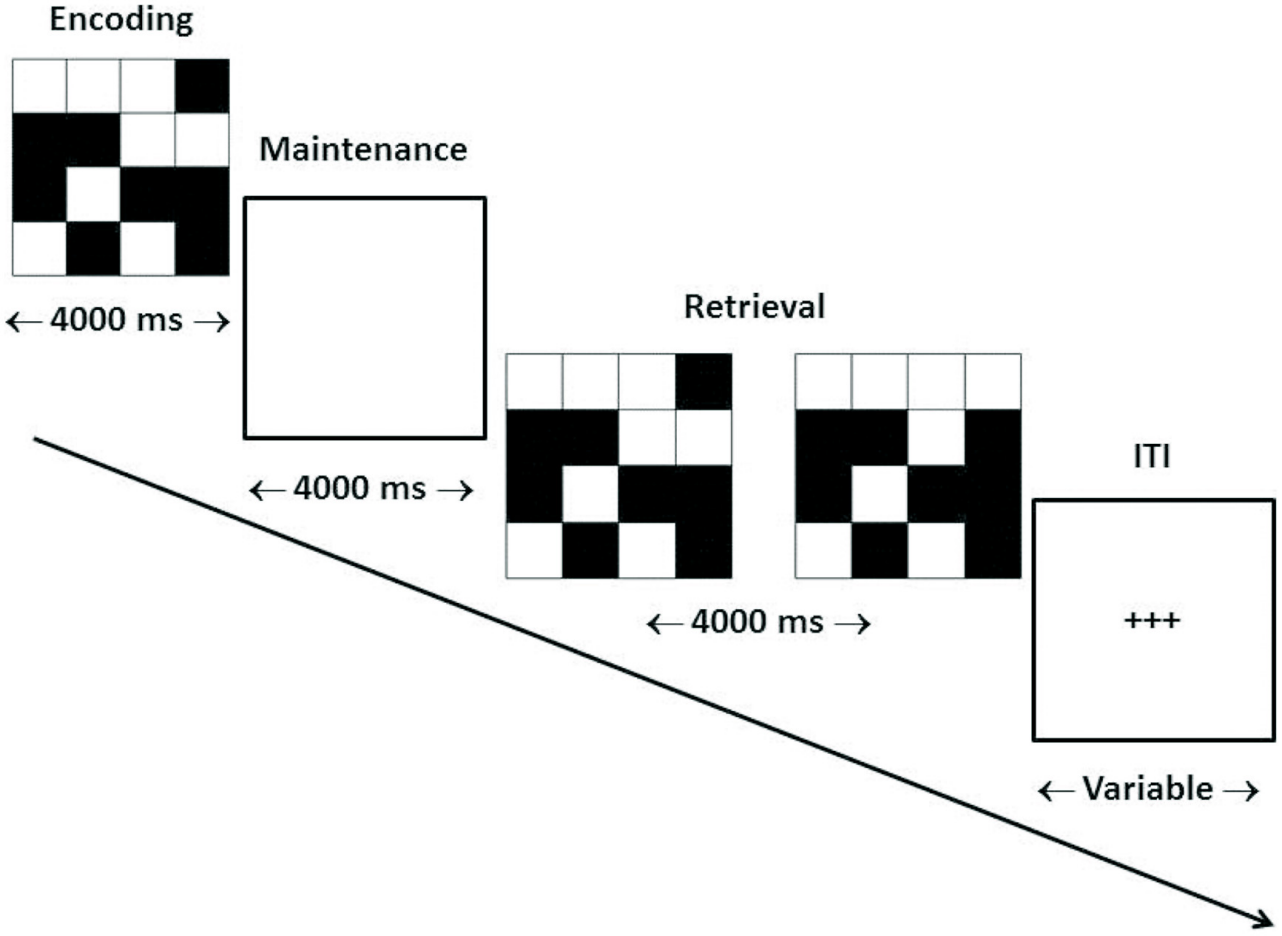
**Trial structure of dMTS**. dMTS, delayed Matching-to-Sample Task. Participants completed 40 trials of identical structure. During *encoding* participants memorized the stimulus. During *maintenance* participants maintained the stimulus in working memory. During *retrieval* participants pressed the button corresponding to the stimulus (left, right) that matched the stimulus presented during *encoding*. The location (left, right) of the matching stimulus was counterbalanced across trials. The ITI varied randomly between 4,000 and 6,000 ms. ITI, inter-trial interval. Arrow indicates direction of trial.

Our target WM task consisted of the delayed matching-to-sample (dMTS) task, a classic measure of short-term visual WM from the animal learning and WM literatures (Miller et al., 1996). dMTS involves the encoding, maintenance, and retrieval of stimulus representations in sequential order (see **Figure 1**). Specifically, during *encoding* participants memorize the stimulus, during *maintenance* they maintain the stimulus in WM, and during *retrieval* they press the button corresponding to the stimulus that matches the stimulus presented during *encoding*. Importantly, both n-back and dMTS are considered to be WM tasks (Rottschy et al., 2012), although as noted above they include different sub-processes. An analysis of n-back and dMTS demonstrates that both engage the maintenance function of WM. Specifically, the n-back task necessitates that stimuli be maintained in WM across presentations so that decisions (match vs. no match) can be made. In turn, in dMTS a stimulus must be maintained for specific delay durations in WM to enable subsequent recognition among the available candidates. We therefore hypothesized that training the maintenance function of WM during n-back would confer an advantage to dMTS performance by virtue of influencing its maintenance phase, because that phase necessitates the maintenance of visual representations in WM.

Importantly, n-back training could also impact the encoding and/or retrieval phases of dMTS because both tasks likely share those sub-processes beyond maintenance alone. For example, although there are explicit and compartmentalized encoding and maintenance phases within dMTS, the updating function inherent in the n-back very likely requires the encoding of memory representations as well as their retrieval for making matching decisions.

In order to pinpoint the locus of transfer-related brain activity, we used fMRI to determine the phase within dMTS wherein activation would distinguish the experimental and control groups. Specifically, if as hypothesized training on the n-back task were to confer an advantage to dMTS performance by virtue of improving the maintenance function within WM, then one should observe a neural difference between the two groups in the maintenance phase. Furthermore, the differences between the two groups during the maintenance phase should be apparent in regions known to underlie delay-period maintenance in visual WM, including primarily the dorsolateral and ventrolateral prefrontal cortex (PFC; BAs 9, 44, 45, and 47), the inferior parietal lobule (BA 40) and adjacent parietal regions (see de Zubicaray et al., 2001; Ranganath et al., 2004). Consistent with the idea that training on the n-back could also be related to variation in brain function in the encoding and retrieval phases of dMTS, we also compared the effect of training (i.e., n-back vs. 4-choice RT) within those two phases.

## Materials and Methods

### Participants

Our protocol was approved by Defence Research and Development Canada’s Human Research Ethics Committee. The 43 participants (35 males, eight females) were neurologically healthy right-handed (Oldfield, 1971) volunteers (*M* = 30.76 years, SD = 9.71) with normal or corrected-to-normal vision. They were assigned randomly to the experimental (*N* = 22) or active control group (*N* = 21). To ensure similar expectations and motivations, participants were not informed about the existence of the two training conditions, or our hypotheses about the differential effects of training on outcome measures of interest (see Boot et al., 2011). There was no significant difference between the two groups in sex [χ^2^ (1) = 0.01, *p* = 0.94], age [*t*(36) = 0.33, *p* = 0.74], or fluid intelligence [*t*(41) = 0.16, *p* = 0.87]—assessed by administering the 18 even or odd items of *Raven’s Advanced Progressive Matrices* (Raven et al., 1998) within a time limit of 10 min (see Jaeggi et al., 2008).

### Materials and Procedure

#### Cognitive Training

All participants completed three 20-min training sessions on separate days, administered using the *Cognitive Test Software* (Grushcow, 2008). Average lag time between successive sessions was 1.21 days (SD = 0.55). Durations and frequencies in WM training studies have varied greatly, ranging from a single 20-min session to 20 h spread over 10 weeks (see Buschkuehl et al., 2012, Table 1; Klingberg, 2010, Table 2). We focused on a short and concentrated training regimen specifically because we were interested in assessing its feasibility as an intervention strategy in applied professional and educational settings.

#### n-back

Participants in the experimental group completed the n-back task. Each session consisted of four blocks—two blocks of 2-back and two blocks of 3-back—administered in alternating order and always starting with 2-back. The stimuli in our variant of the n-back were letters. No vowels were used in the task, and we only used a subset of consonants (X, G, H, K, P, Q, S, and W). We did not control for interference lures. Each block contained 150 trials. On 50 trials within each block the presented letter matched the target letter presented two or three positions earlier in the sequence (depending on the block), whereas on the remaining 100 trials it did not. Each letter was presented for 500 ms. Inter-stimulus interval (ISI) was a blank screen presented for 2500 ms. Participants pressed the spacebar when they detected a match.

#### 4-choice RT

Participants in the active control group completed the 4-choice RT task (Dollins et al., 1993). On each trial of this task, one of four adjacent locations on the computer screen was highlighted randomly. Participants pressed one of four keys corresponding to the highlighted location. We selected this task to control for task engagement not involving a WM task. Participants completed 420 trials per session. Based on normative data collected in our lab from the same population using the same task (Nakashima et al., 2011), we expected accuracy to be at ceiling across the three sessions.

#### dMTS

Participants completed the dMTS in the fMRI scanner 3.29 days (SD = 1.11) after the last training session (see **Figure 1**).

#### fMRI Acquisition

A 3-Tesla MR scanner with an 8-channel head coil (Discovery MR750, 22.0 software, GE Healthcare, Waukesha, WI, USA) was used to acquire T1 anatomical volume images (0.86 mm × 0.86 mm × 1.0 mm voxels). For functional imaging, T2^∗^-weighted gradient echo spiral-in/out acquisitions were used to produce 26 contiguous 5 mm thick axial slices [repetition time (TR) = 2000 ms; echo time (TE) = 30 ms; flip angle (FA) = 70°; field of view (FOV) = 200 mm; 64 × 64 matrix; voxel dimensions = 3.1 mm × 3.1 mm × 5.0 mm], positioned to cover the whole brain. The first five volumes were discarded to allow for T1 equilibration effects. The number of volumes acquired was 354.

#### fMRI Analysis

Data were analyzed using Statistical Parametric Mapping (SPM8). Head movement was less than 2 mm. All functional volumes were spatially realigned to the first volume. A mean image created from realigned volumes was spatially normalized to the MNI EPI brain template using non-linear basis functions. The derived spatial transformation was applied to the realigned T2^∗^ volumes, and spatially smoothed with an 8 mm full-width at half-maximum isotropic Gaussian kernel. Time series across each voxel were high-pass filtered with a cut-off of 128 s, using cosine functions to remove section-specific low frequency drifts in the BOLD signal. Condition effects at each voxel were estimated according to the GLM and regionally specific effects compared using linear contrasts. The BOLD signal was modeled as a box-car, convolved with a canonical hemodynamic response function.

We applied a combination of voxel-height and cluster extent correction for multiple comparisons using AlphaSim (http://afni.nimh.nih.gov/pub/dist/doc/manual/AlphaSim.pdf) incorporated in REST (Song et al., 2011). Whereas originally AlphaSim was developed for use within the Analysis of Functional Neuroimages (AFNI) software (Cox, 1996), REST enables one to conduct the same analysis on a Windows platform using SPM masks. AlphaSim takes into account the size of the search space and the estimated smoothness, and using Monte Carlo simulations generates probability estimates of a random field of noise, producing a cluster of voxels of a given size for a set of voxels passing a given voxel-wise *p*-value threshold. Using a random-effects analysis, we report activations that survived *p* < 0.05—corrected for multiple comparisons (FWE) within the avg152T2.nii whole-brain mask from the SPM toolbox. The real smoothness in the three directions was estimated from the residuals (FWHMx = 11.699 mm, FWHMy = 11.869 mm, FWHMz = 10.992 mm). Within our mask, the Monte Carlo simulations determined that a FWE-corrected false-positive probability of *p* < 0.05 was achieved using a voxel-wise threshold of *p* < 0.005 combined with a spatial extent threshold of 249 voxels.

## Results

### Cognitive Training

For the experimental group we conducted a repeated-measures ANOVA with session (1, 2, and 3) and level (2-back, 3-back) as within-subjects variables. The key dependent variable was *d*′ (sensitivity; Stanislaw and Todorov, 1999; see Kane et al., 2007). When *d*′ is positive (and high), participants are considered to display good sensitivity, whereas when *d*′ is negative participants are incorrectly judging matches as mismatches and vice versa. In addition, we also investigated the effects of the two independent variables on the criterion—defined as the value of the decision variable deemed sufficiently high to determine that there is a match. A liberal value for the criterion biases the participant toward responding that there is a match, whereas a conservative value biases the participant toward responding that there is no match.

**FIGURE 2:**
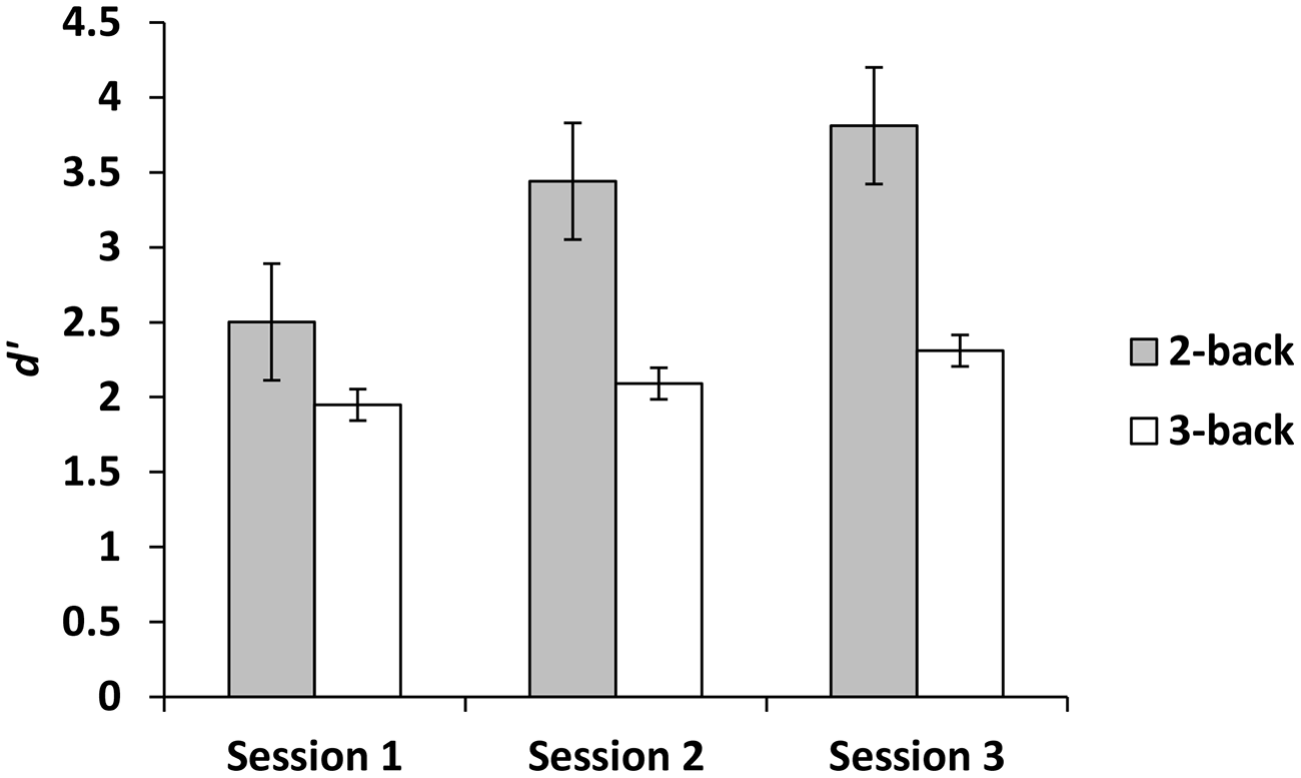
**The effects of session and level on n-back performance during training**.

For the experimental group, there was a main effect for session, demonstrating that *d*′ improved across sessions, *F*(2,42) = 10.50, *p* < 0.001, $\eta_{p}^{2}$ = 0.33. Paired comparisons demonstrated that compared to session 1, *d*′ was higher at sessions 2 and 3. There was no difference between sessions 2 and 3 (*p* = 0.10). There was also a main effect for level, demonstrating that *d*′ was greater on 2-back than 3-back, *F*(1,21) = 25.05, *p* < 0.001, $\eta_{p}^{2}$ = 0.54. In addition, there was a session × level interaction such that across three sessions *d*′ improved more for 2-back than 3-back, *F*(2,42) = 5.90, *p* < 0.01, $\eta_{p}^{2}$ = 0.22 (**Figure 2**). In contrast, when we focused on the criterion as the dependent variable, the effects of session, level and the session × level interaction were not significant (all *p*s ≥ 0.99).

For the active control group we conducted an ANOVA with session (1, 2, and 3) as the within-subjects variable, and accuracy as the dependent variable. As predicted (see Nakashima et al., 2011), performance was at ceiling across sessions 1 (*M* = 96.57%, SD = 3.10), 2 (*M* = 95.93%, SD = 4.14), and 3 (*M* = 96.86%, SD = 2.24), *F*(2,38) = 1.48, *p* = 0.25, $\eta_{p}^{2}$ = 0.07. We conducted an additional ANOVA with session (1, 2, and 3) as the within-subjects variable, and RT as the dependent variable. There was a main effect such that RT decreased across the first (*M* = 0.45 s, SD = 0.06), second (*M* = 0.42 s, SD = 0.05) and third sessions (*M* = 0.41 s, SD = 0.05), *F*(2,38) = 15.13, *p* = < 0.001, $\eta_{p}^{2}$ = 0.44.

### dMTS

**FIGURE 3:**
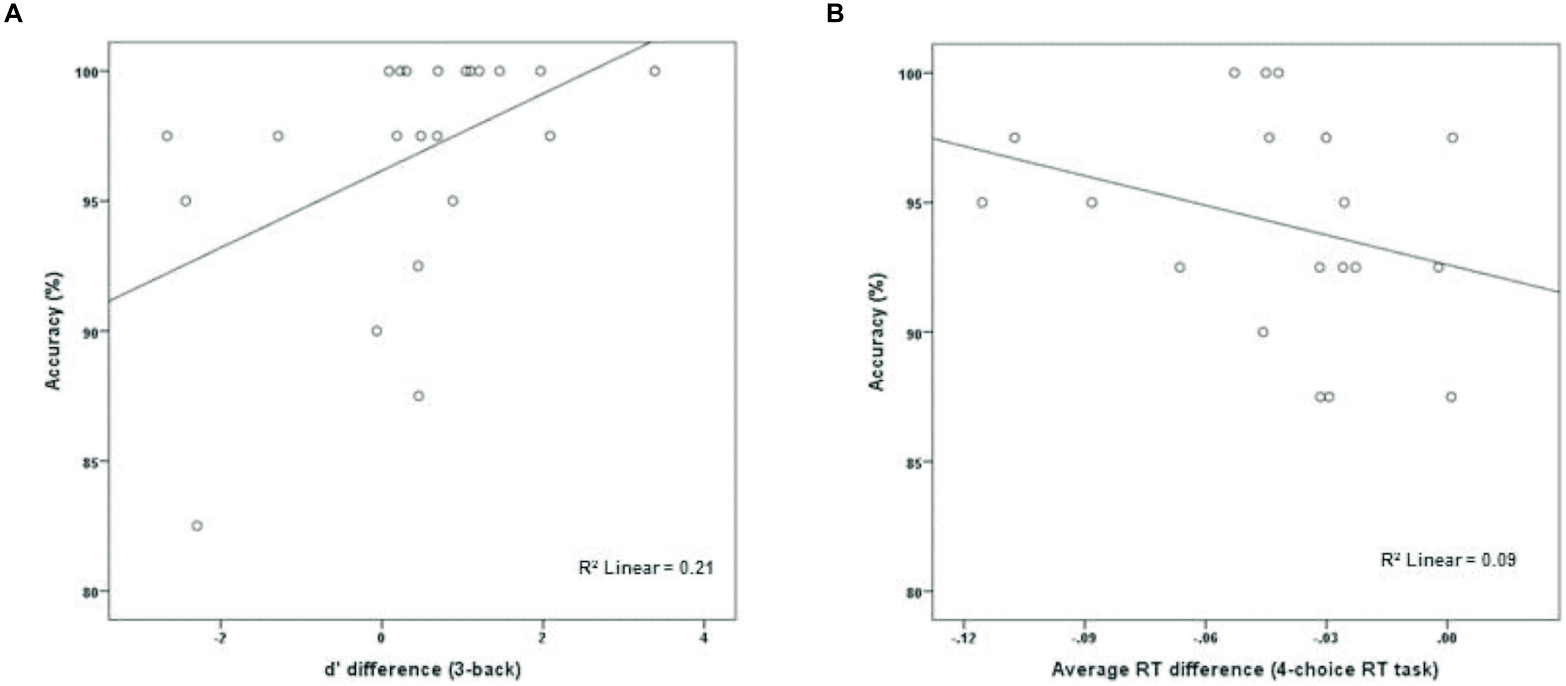
**The relationship between degree of training-related improvement in **(A)** n-back and **(B)** 4-choice RT and dMTS performance**. Whereas the degree of training-related improvement in n-back predicted dMTS performance, degree of improvement in 4-choice RT did not (see text).

The experimental group (*M* = 96.70%, SD = 4.72) performed marginally better than the active control group (*M* = 93.81%, SD = 5.22) on dMTS, *t*(41) = 1.91, *p* = 0.06, Cohen’s *d* = 0.58. To directly test whether performance on dMTS would be a function of improvement in training on the n-back, for the experimental group we computed a new variable that was the difference in *d′* between session 1 and session 3 (*d′*_difference_ = *d′*_session 3_ -*d′*_session 1_)—separately for 2-back and 3-back. Next, we regressed accuracy (%) in dMTS performance onto *d′*_difference_. The results demonstrated that degree of improvement in 2-back was unrelated to dMTS performance, β = 0.31, *p* = 0.16. In contrast, degree of improvement in 3-back predicted variation in dMTS performance, β = 0.46, *p* = < 0.05. This result demonstrates that the degree of improvement in 3-back is a significant factor in dMTS performance. In fact, improvement in 3-back performance during training accounted for 21% of the variance in dMTS performance (**Figure 3A**).

To determine whether the degree of improvement in 4-choice RT was predictive of dMTS performance amongst participants in the control group, we computed a new variable that was the difference in RT between session 1 and session 3 (RT_difference_ = RT_session 3_ - RT_session 1_). Next, we regressed accuracy (%) in dMTS performance onto RT_difference_. Importantly, only 19 data points (rather than 21) were included in this analysis because one participant failed to complete the third session of training, and another data point was excluded because it was an outlier—determined by its deviation from the means of both distributions by approximately 3 SDs (see Wainer, 1976). Degree of improvement in RT was unrelated to dMTS performance, β = −0.30, *p* = 0.21 (**Figure 3B**).

### fMRI

**FIGURE 4:**
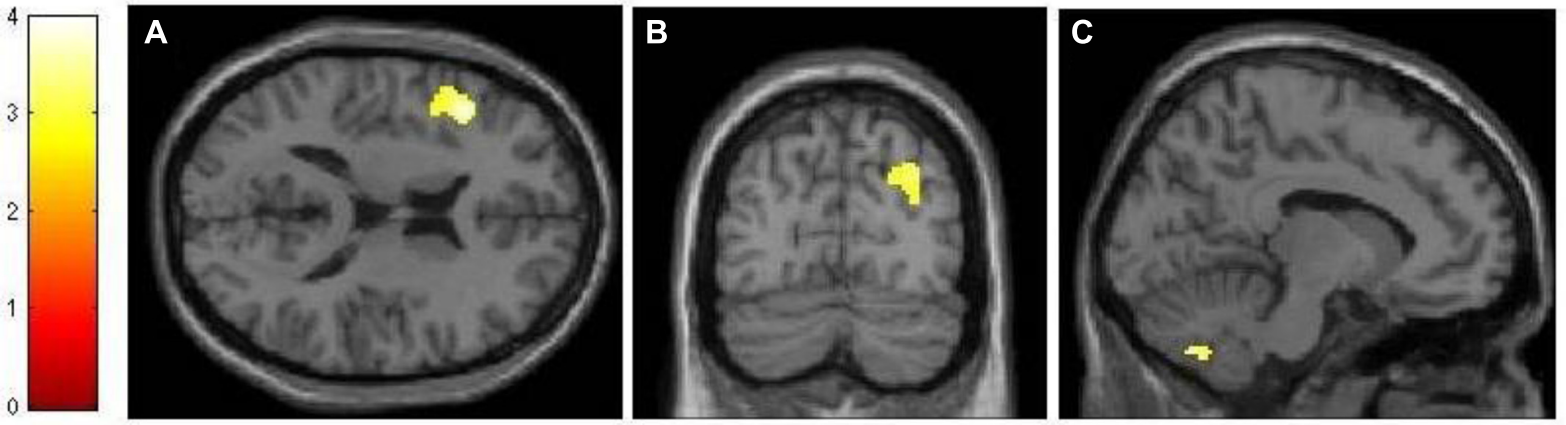
**Neural differences between experimental and control conditions during the maintenance phase of dMTS**. There was greater activation in left IFG **(A)**, right PPC **(B)**, and cerebellum **(C)** in the experimental than control group during maintenance. SPMs rendered into standard stereotactic space and superimposed on to transverse **(A)**, coronal **(B)**, and saggital **(C)** MRI in standard space. Bar represents the corresponding *T*-score. dMTS, delayed Matching-to-Sample Task, IFG, inferior frontal gyrus, PPC, posterior parietal cortex.

Using an event-related design, we specified six regressors corresponding to (1) encoding, (2) maintenance, (3) retrieval, (4) ISI, (5) ITI, and (6) motor response. ISI and motor response were modeled out of the analyses by assigning weights of 0 to their corresponding regressors in all analyses. **Table 1** lists the regions activated in the encoding (-ITI), maintenance (-ITI), and retrieval (-ITI) phases of dMTS *across all participants*. An independent-samples *t*-test demonstrated greater activation in the n-back than active control group in the maintenance phase in the left inferior frontal gyrus (IFG; *T* = 3.97, *k_E_* = 389, *x* = −44, *y* = 22, *z* = 16), the right posterior parietal cortex (PPC; *T* = 3.30, *k_E_* = 299, *x* = 32, *y* = −74, *z* = 36), and the cerebellum (*T* = 3.56, *k_E_* = 277, *x* = −10, *y* = −68, *z* = −42; **Figure 4**). Neither the reverse contrast nor the contrasts in either direction involving the encoding or retrieval phase revealed any significant difference between the two groups. In other words, the difference in brain activation between the n-back and active control groups was limited exclusively to the maintenance phase of dMTS.

**Table 1:** Coordinates for the observed activations in the encoding, maintenance, and retrieval phases of delayed matching-to-sample task (dMTS; vs. rest) across all participants.

Contrast	Structure	*x*	*y*	*z*	*T-score*
Encoding-ITI	Precuneus	−16	−68	50	13.52
	Precuneus	24	−68	46	11.89
	Precentral gyrus	−46	−6	46	12.27
	Parahippocampus	38	−14	−30	4.90
Maintenance-ITI	Precuneus	−24	−64	50	13.25
	Inferior parietal lobe	−36	−44	40	12.81
	Superior parietal lobe	12	−64	62	11.70
Retrieval-ITI	Anterior insula	32	26	−2	11.52
	Anterior insula	−28	24	0	9.68
	Cerebellum	0	−50	−36	5.87

*The coordinates are reported in MNI space. All reported activation survived whole-brain family-wise error (FWE) correction (*p* < 0.05) as implemented by AlphaSim in REST (Song et al., 2011).*

**FIGURE 5:**
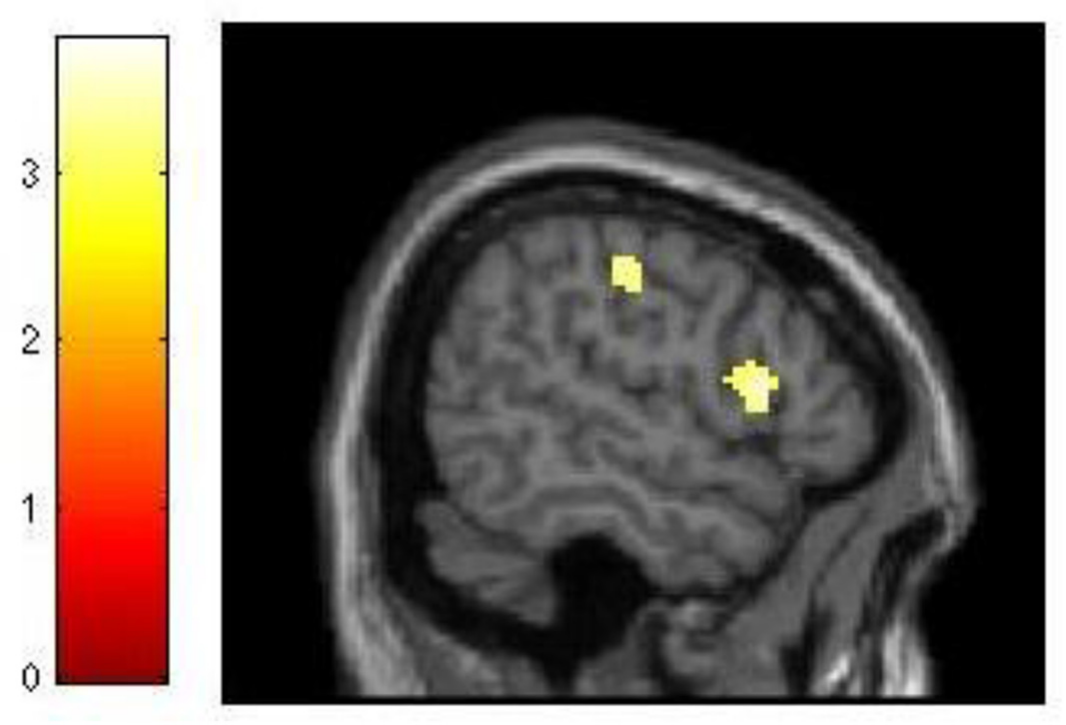
**Relationship between training-related improvement in 3-back and brain activation in the maintenance phase of dMTS**. Activation in right lateral PFC and motor cortex covaried with degree of training-related improvement in 3-back. SPM rendered into standard stereotactic space and superimposed on saggital MRI in standard space. Bar represents the corresponding *T*-score. dMTS, delayed Matching-to-Sample Task.

The analysis of our behavioral data had demonstrated that improvement in 3-back performance during training accounted for 21% of the variance in dMTS performance (**Figure 3A**). To explore this effect at the neural level, we conducted three separate regression analyses to see whether difference in *d′* for 3-back (*d′*_difference_ = *d′*_session 3_ -*d′*_session 1_) would covary with brain activation during (1) encoding, (2) maintenance, and (3) retrieval. The results demonstrated that brain activation did not covary in relation to *d′*_difference_ during encoding or retrieval. In contrast, during the maintenance phase brain activation in right lateral PFC (*T* = 3.68, *k_E_* = 260, *x* = 56, *y* = 16, *z* = 10) and motor cortex (*T* = 3.78, *k_E_* = 421, *x* = 46, *y* = −22, *z* = 44) covaried with *d′*_difference_ (**Figure 5**).

**FIGURE 6:**
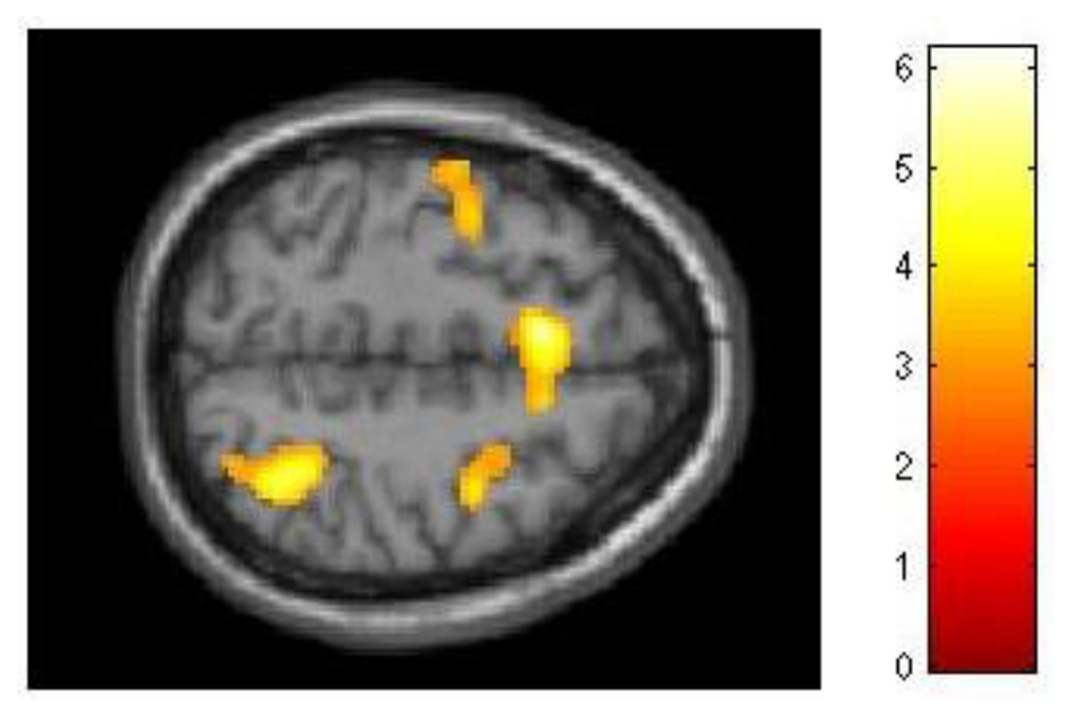
**Relationship between training-related improvement in 2-back and brain activation in the maintenance phase of dMTS**. Activation in a distributed network including the superior parietal lobe and middle frontal gyrus (left, right, medial) covaried with degree of training-related improvement in 2-back. SPM rendered into standard stereotactic space and superimposed on transverse MRI in standard space. Bar represents the corresponding *T*-score. dMTS, delayed Matching-to-Sample Task.

Although our behavioral data had demonstrated that for the experimental group improvement in 2-back performance was unrelated to dMTS performance, we nevertheless explored this effect at the neural level. As with 3-back, we conducted three separate regression analyses to see whether difference in *d′* for 2-back (*d′*_difference_ = *d′*_session 3_ -*d′*_session 1_) would covary with brain activation during (1) encoding, (2) maintenance, and (3) retrieval. Demonstrating a pattern similar to 3-back, brain activation did not covary in relation to *d′*_difference_ during encoding or retrieval. However, during the maintenance phase *d′*_difference_ covaried with activation in a distributed network in the brain, including three locations in right (*T* = 6.18, *k_E_* = 2164, *x* = 44, *y* = 4, *z* = 28), left (*T* = 4.66, *k_E_* = 2161, *x* = −42, *y* = 2, *z* = 16), and medial (*T* = 5.51, *k_E_* = 982, *x* = −8, *y* = 14, *z* = 52) middle frontal gyrus (BA 6), left lateral PFC (*T* = 4.62, *k_E_* = 940, *x* = −42, *y* = 24, *z* = 6), right superior parietal lobule (*T* = 5.06, *k_E_* = 765, *x* = 36, *y* = −56, *z* = 54), right cingulate (*T* = 4.57, *k_E_* = 358, *x* = 12, *y* = −10, *z* = 36), right extrastriate cortex (*T* = 4.68, *k_E_* = 669, *x* = 36, *y* = -76, *z* = 20), and three locations in left (*T* = 5.52, *k_E_* = 2059, *x* = −18, *y* = −74, *z* = −18; *T* = 4.60, *k_E_* = 1263, *x* = −10, *y* = −50, *z* = −48) and right (*T* = 3.80, *k_E_* = 504, *x* = 28, *y* = −54, *z* = −32) cerebellum (**Figure 6**).

Finally, although our behavioral data had demonstrated that for the control group improvement in the 4-choice RT task was unrelated to dMTS performance, we nevertheless explored this effect at the neural level as well. Specifically, we conducted three separate regression analyses to see whether difference in RT in the 4-choice RT task (RT_difference_ = RT_session 3_ - RT_session 1_) would covary with brain activation during (1) encoding, (2) maintenance, and (3) retrieval. Our results demonstrated that there was no relationship between brain activation and RT_difference_ during encoding, maintenance or the retrieval phase.

## Discussion

The n-back group performed marginally better than the active control group on dMTS, registering a medium effect size (Cohen, 1988). Importantly, although participants in the experimental condition were more likely to exhibit improvement across the three training sessions in the 2-back level than the 3-back level (**Figure 2**), it was their degree of improvement in the 3-back level that predicted variation in dMTS performance, accounting for 21% of the observed variance in dMTS performance (**Figure 3A**). Critically, degree of improvement in the 4-choice RT task in the control condition was unrelated to dMTS performance or its neural correlates, despite the fact that both are visuospatial tasks. These results demonstrate a dissociation between how training-related improvement in a WM task vs. a non-WM task is related to a target WM task. More specifically, they suggest that although performance on relatively easier levels of n-back is more likely to improve within three brief practice sessions, it is improvement in the more difficult levels that is more likely to be positively related to performance on target tasks drawing on the same capacity.

In addition, the neural difference between the two groups was only apparent during the maintenance phase of dMTS, and localized to the left IFG, right PPC and the cerebellum. Sustained activation in the PFC has been related to maintenance in memory (Fuster, 1991). Indeed, IFG activation has been shown to be involved in the maintenance phase of the delayed non-matching-to-sample task (de Zubicaray et al., 2001). This is consistent with the involvement of the ventrolateral regions of the left PFC in delay-period maintenance in visual WM tasks (Ranganath et al., 2004). In addition, posterior parietal regions have been shown to contribute to various aspects of visual short-term mnemonic function including maintenance (Munk et al., 2002) and active maintenance of information in WM (Cohen et al., 1997). In fact, PPC activity has been shown to predict individual differences in visual short-term memory capacity (Todd and Marois, 2005; see also Todd and Marois, 2004). Our neural results suggest that the effects of n-back training on transfer-related brain function in dMTS are likely to be observed in regions that underlie capacities enhanced during training, and subsequently recruited by the untrained task. Our behavioral task analysis had led us to believe that n-back training would likely benefit dMTS performance because both tasks tax the maintenance function in WM, among others. Our neural results are generally consistent with this idea, although further experimentation is needed to determine that the regions distinguishing the two groups during maintenance indeed underlie transfer from n-back to dMTS.

Because training-related improvement in 3-back predicted dMTS performance (**Figure 3A**), we explored this effect at the neural level. Our results revealed that during the maintenance phase of dMTS, brain activation in right lateral PFC and motor cortex covaried with training-related improvement in 3-back (**Figure 5**). This region of the lateral PFC corresponds to Brodmann Area 44, and has been shown to be involved in both the storage and manipulation aspects of WM (see Wager and Smith, 2003). Our results suggest that this region is sensitive to training-related changes in relation to 3-back, and could be a region shared by both the n-back and dMTS for maintenance in WM.

In addition, although behaviourally training-related improvement in 2-back was unrelated to dMTS performance, our analyses of fMRI data demonstrated that during the maintenance phase of dMTS brain activation in a distributed network including the middle frontal gyrus, lateral PFC, superior parietal lobule, cingulate, extrastriate cortex, and the cerebellum covaried with training-related improvement in 2-back (**Figure 6**). Within this network, the frontal and parietal regions represent well-established nodes in the fronto-parietal WM network (Petrides, 2005; D‘Esposito, 2008). Although these results demonstrate that brain activation during the maintenance phase of dMTS was modulated by the degree of training-related improvement in 2-back, care must be exercised in interpreting this finding given the absence of a corresponding behavioral effect (**Figure 3B**).

### Limitations

Our results must be considered preliminary because our study had a number of limitations. First, our design involved randomly assigning participants to two treatment conditions, and subsequently measuring differences between the two groups on an outcome measure (i.e., dMTS) following training. As such, our results are correlational, and we cannot draw causal inferences. In addition, although the degree of training-related improvement in 3-back predicted and accounted for 21% of the variance in dMTS performance (**Figure 3A**), gain data alone cannot be used as evidence for inferring transfer effects (Tidwell et al., 2014). Rather, there is reason to further explore the possibility of a causal link between n-back training and performance on other WM tasks, including dMTS.

Second, our active control condition was meant to control for task engagement only—defined by identical frequency and duration of training. Although in WM training studies active control conditions are preferable to passive control conditions (Shipstead et al., 2010), it would be better still to include both types of control conditions in a given design. Particularly desirable would be to use control conditions that enable one to isolate specific components of training that are believed to be related to transfer to dMTS performance (e.g., updating, maintenance, etc.). The present results lay the groundwork for implementing such a design feature in future studies, perhaps comparing different types of WM training that tax different aspects of WM function.

Third, although the durations and frequencies of training sessions in WM training studies have ranged greatly in the past, ranging from one 20- or 30-min session to 20 h spread over 10 weeks (see Buschkuehl et al., 2012, Table 1; Klingberg, 2010, Table 2), our WM training intervention was relatively short and involved a non-adaptive WM task. Future studies would benefit from implementing an adaptive WM task, possibly administered in the context of more frequent training sessions.

Fourth, it is likely that there was a ceiling effect associated with our outcome measure (dMTS). In turn, this might have made it more difficult to observe differences between the two training conditions on this task, given that there was less room for improvement. There are at least two ways to increase the difficulty level on dMTS. First, on each trial we used a stimulus consisting of a 4 × 4 matrix (**Figure 1**). Doubling the matrix dimensions (i.e., 8 × 8) reduces average accuracy rates to around 70% (Nakashima et al., 2011). Second, whereas we used a fixed delay period, dMTS paradigms can incorporate variable delay periods. This will enable one to analyze differences in performance as a function of varying delay periods. These modifications can be incorporated in future studies.

## Conclusion

Our results demonstrated that a group training on the n-back task performed marginally better than an active control group on dMTS. Although the n-back group improved more on 2-back than 3-back across three training sessions, it was improvement in 3-back that predicted and accounted for 21% of the variance in dMTS performance. There was no relationship between training-related gains and dMTS performance in the control group. At the neural level, the n-back group exhibited greater activation in the left IFG, right PPC and the cerebellum during the maintenance phase within dMTS. In addition, degree of improvement in 3-back covaried with brain activation in the right lateral prefrontal and motor cortices during the maintenance phase of dMTS, as did the degree of improvement in 2-back and activation in a distributed network including fronto-parietal WM nodes. In contrast, in the control group no relationship was observed between degree of improvement on the 4-choice RT task and dMTS performance. Combined, our results suggest that n-back training is more closely associated with dMTS performance than training on a task that does not tax WM.

## Conflict of Interest Statement

The authors declare that the research was conducted in the absence of any commercial or financial relationships that could be construed as a potential conflict of interest.

## References

[B1] Boot W. R. Blakely D. P. Simons D. J. (2011). Do action video games improve perception and cognition? *Front. Psychol.* 2:226 10.3389/fpsyg.2011.00226 PMC3171788 21949513

[B2] Buschkuehl M. Jaeggi S. M. Jonides J. (2012). Neuronal effects following working memory training. *Dev. Cogn. Neurosci.* 2(Suppl. 1), S167–S179. 10.1016/j.dcn.2011.10.001 22682905 PMC6987667

[B3] Cohen J. (1988). *Statistical Power Analysis for the Behavioral Sciences*. Hillsdale, NJ: Lawrence Erlbaum Associates.

[B4] Cohen J. D. Perlstein W. M. Braver T. S. Nystrom L. E. Noll D. C. Jonides J. (1997). Temporal dynamics of brain activation during a working memory task. *Nature* 386 604–608. 10.1038/386604a0 9121583

[B5] Conway A. R. Kane M. J. Bunting M. F. Hambrick D. Z. Wilhelm O. Engle R. W. (2005). Working memory span tasks: a methodological review and user’s guide. *Psychon. Bull. Rev.* 12 769–786. 10.3758/BF03196772 16523997

[B6] Cox R. W. (1996). AFNI: software for analysis and visualization of functional magnetic resonance neuroimages. *Comput. Biomed. Res.* 29 162–173. 10.1006/cbmr.1996.0014 8812068

[B7] Dahlin E. Neely A. S. Larsson A. Backman L. Nyberg L. (2008). Transfer of learning after updating training mediated by the striatum. *Science* 320 1510–1512. 10.1126/science.1155466 18556560

[B8] D‘Esposito M. (2008). “Working memory,” in *Handbook of Clinical Neurology: Neuropsychology and Behavioral Neurology*, eds Goldenberg G. Miller B. (Amsterdam: Elsevier), 237–247.

[B9] de Zubicaray G. I. Mcmahon K. Wilson S. J. Muthiah S. (2001). Brain activity during the encoding, retention, and retrieval of stimulus representations. *Learn. Mem.* 8 243–251. 10.1101/lm.40301 11584070 PMC311385

[B10] Dollins A. B. Lynch H. J. Wurtman R. J. Deng M. H. Kischka K. U. Gleason R. E. (1993). Effect of pharmacological daytime doses of melatonin on human mood and performance. *Psychopharmacology (Berl.)* 112 490–496. 10.1007/BF02244899 7871062

[B11] Fuster J. M. (1991). The prefrontal cortex and its relation to behavior. *Prog. Brain Res.* 87 201–211. 10.1016/j.pscychresns.2009.03.012 1907745

[B12] Grushcow M. (2008). *Cognitive Test Software [Computer Softwarew]*. North York, ON: NTT Systems Inc.

[B13] Harrison T. L. Shipstead Z. Hicks K. L. Hambrick D. Z. Redick T. S. Engle R. W. (2013). Working memory training may increase working memory capacity but not fluid intelligence. *Psychol. Sci.* 24 2409–2419. 10.1177/0956797613492984 24091548

[B14] Jaeggi S. M. Buschkuehl M. Jonides J. Perrig W. J. (2008). Improving fluid intelligence with training on working memory. *Proc. Natl. Acad. Sci. U.S.A.* 105 6829–6833. 10.1073/pnas.0801268105 18443283 PMC2383929

[B15] Kane M. J. Conway A. R. Miura T. K. Colflesh G. J. (2007). Working memory, attention control, and the N-back task: a question of construct validity. *J. Exp. Psychol. Learn. Mem. Cogn.* 33 615–622. 10.1037/0278-7393.33.3.615 17470009

[B16] Kane M. J. Engle R. W. (2002). The role of prefrontal cortex in working-memory capacity, executive attention, and general fluid intelligence: an individual-differences perspective. *Psychon. Bull. Rev.* 9 637–671. 10.3758/BF03196323 12613671

[B17] Karbach J. Kray J. (2009). How useful is executive control training? Age differences in near and far transfer of task-switching training. *Dev. Sci.* 12 978–990. 10.1111/j.1467-7687.2009.00846.x 19840052

[B18] Klingberg T. (2010). Training and plasticity of working memory. *Trends Cogn. Sci.* 14 317–324. 10.1016/j.tics.2010.05.002 20630350

[B19] Melby-Lervåg M. Hulme C. (2013). Is working memory training effective? A meta-analytic review. *Dev. Psychol.* 49 270–291. 10.1037/a0028228 22612437

[B20] Miller E. K. Erickson C. A. Desimone R. (1996). Neural mechanisms of visual working memory in prefrontal cortex of the macaque. *J. Neurosci.* 16 5154–5167. 8756444 10.1523/JNEUROSCI.16-16-05154.1996 PMC6579322

[B21] Morrison A. B. Chein J. M. (2011). Does working memory training work? The promise and challenges of enhancing cognition by training working memory. *Psychon. Bull. Rev.* 18 46–60. 10.3758/s13423-010-0034-30 21327348

[B22] Munk M. H. Linden D. E. Muckli L. Lanfermann H. Zanella F. E. Singer W. (2002). Distributed cortical systems in visual short-term memory revealed by event-related functional magnetic resonance imaging. *Cereb. Cortex* 12 866–876. 10.1093/cercor/12.8.866 12122035

[B23] Nakashima A. Vartanian O. Bouak F. Hofer K. Cheung B. (2011). “A test battery for the assessment of psychological and physiological performance following primary blast wave exposure,” in *Proceedings of the Military and Veterans Health Research Forum* eds Aiken A. B. Bélanger S. A. H. (Kingston, ON: Canadian Defence Academy Press), 134–153.

[B24] Oldfield R. C. (1971). The assessment and analysis of handedness: the Edinburgh inventory. *Neuropsychologia* 9 97–113. 10.1016/0028-3932(71)90067-4 5146491

[B25] Persson J. Welsh K. M. Jonides J. Reuter-Lorenz P. A. (2007). Cognitive fatigue of executive processes: interaction between interference resolution tasks. *Neuropsychologia* 45 1571–1579. 10.1016/j.neuropsychologia.2006.12.007 17227678 PMC1876692

[B26] Petrides M. (2005). Lateral prefrontal cortex: architectonic and functional organization. *Philos. Trans. R. Soc. Lond. B Biol. Sci.* 360 781–795. 10.1098/rstb.2005.1631 15937012 PMC1569489

[B27] Ranganath C. Cohen M. X. Dam C. D’esposito M. (2004). Inferior temporal, prefrontal, and hippocampal contributions to visual working memory maintenance and associative memory retrieval. *J. Neurosci.* 24 3917–3925. 10.1523/JNEUROSCI.5053-03.2004 15102907 PMC6729418

[B28] Raven J. Raven J. C. Court J. H. (1998). *Raven Manual: Section 4. Advanced Progressive Matrices.* Oxford: Oxford Psychologists Press.

[B29] Rottschy C. Langner R. Dogan I. Reetz K. Laird A. R. Schulz J. B. (2012). Modelling neural correlates of working memory: a coordinate-based meta-analysis. *Neuroimage* 60 830–846. 10.1016/j.neuroimage.2011.11.050 22178808 PMC3288533

[B30] Salminen T. Frensch P. Strobach T. Schubert T. (2015). Age-specific differences of dual n-back training. *Neuropsychol. Dev. Cogn. B Aging Neuropsychol. Cogn.* 1–22. 10.1080/13825585.2015.1031723 [Epub ahead of print]. 25867501

[B31] Salminen T. Strobach T. Schubert T. (2012). On the impacts of working memory training on executive functioning. *Front. Hum. Neurosci.* 6:166 10.3389/fnhum.2012.00166 PMC3368385 22685428

[B32] Shipstead Z. Redick T. S. Engle R. W. (2010). Does working memory training generalize? *Psychol. Belg.* 50 245–276. 10.5334/pb-50-3-4-245

[B33] Song X. W. Dong Z. Y. Long X. Y. Li S. F. Zuo X. N. Zhu C. Z. (2011). REST: a toolkit for resting-state functional magnetic resonance imaging data processing. *PLoS ONE* 6:e25031 10.1371/journal.pone.0025031 PMC3176805 21949842

[B34] Sprenger A. M. Atkins S. M. Bolger D. J. Harbison J. I. Novick J. M. Chrabaszcz J. S. (2013). Training working memory: limits of transfer. *Intelligence* 41 638–663. 10.1016/j.intell.2013.07.013

[B35] Stanislaw H. Todorov N. (1999). Calculation of signal detection theory measures. *Behav. Res. Methods Instrum. Comput.* 31 137–149. 10.3758/bf03207704 10495845

[B36] Tidwell J. W. Dougherty M. R. Chrabaszcz J. R. Thomas R. P. Mendoza J. L. (2014). What counts as evidence for working memory training? Problems with correlated gains and dichotomization. *Psychon. Bull. Rev.* 21 620–628. 10.3758/s13423-013-0560-567 24307249

[B37] Todd J. J. Marois R. (2004). Capacity limit of visual short-term memory in human posterior parietal cortex. *Nature* 428 751–754. 10.1038/nature02466 15085133

[B38] Todd J. J. Marois R. (2005). Posterior parietal cortex activity predicts individual differences in visual short-term memory capacity. *Cogn. Affect. Behav. Neurosci.* 5 144–155. 10.3758/cabn.5.2.144 16180621

[B39] Wager T. D. Smith E. E. (2003). Neuroimaging studies of working memory: a meta-analysis. *Cogn. Affect. Behav. Neurosci.* 3 255–274. 10.3758/CABN.3.4.255 15040547

[B40] Wainer H. (1976). Robust statistics: a survey and some prescriptions. *J. Educ. Stat.* 1 285–312. 10.2307/1164985

