# Supplementary figures and images for: Transfer of training from one working memory task to another: behavioural and neural evidence
Source: Front Syst Neurosci. 2015 Jun 2;9:86. doi: 10.3389/fnsys.2015.00086 (PMC4451342; doi:10.3389/fnsys.2015.00086)

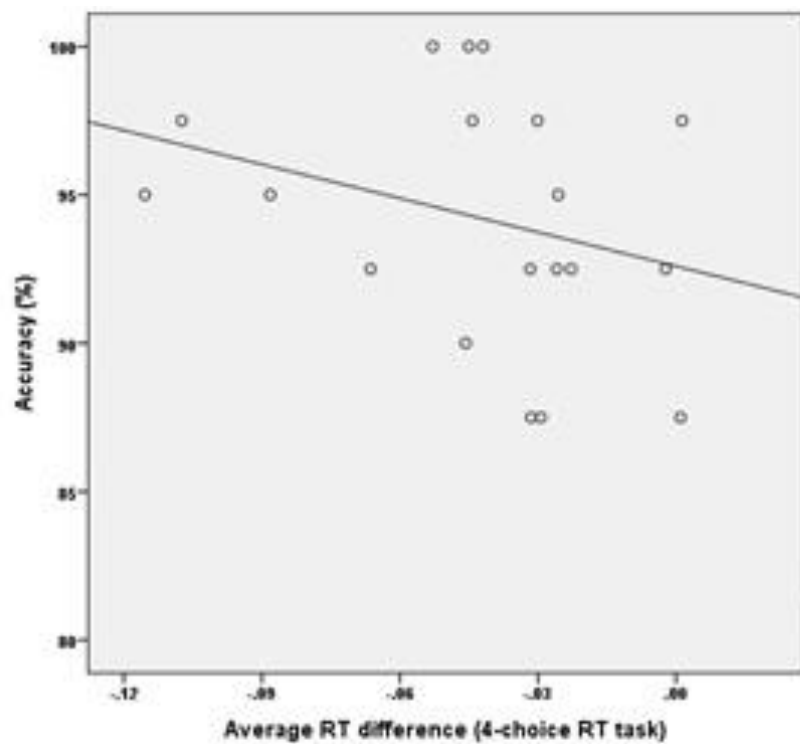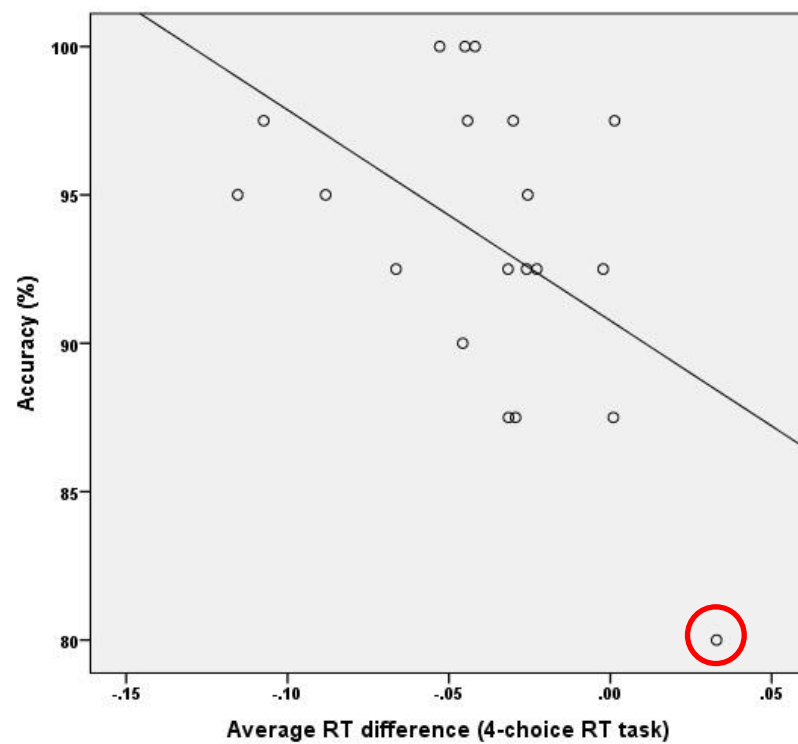

Supplement: Supplementary file 1 [file Image_1.PDF]
